# A comparison of stone-based and diamond strip approaches for adjusting proximal contact tightness in zirconia and PFM crowns

**DOI:** 10.1186/s12903-025-05829-2

**Published:** 2025-04-24

**Authors:** Daniel S. Kim, Soo-Yeon Yoo, Jang-Hyun Kim, Dong-Ju Shin, Ji-Man Park

**Affiliations:** 1 Private practice, Vancouver, USA; 2 Department of Prosthodontics & Dental Research Institute, School of Dentistry, Seoul National University Dental Hospital, Seoul National University, Seoul, Korea; 3 Clinical Researcher, Department of Prosthodontics & Dental Research Institute, School of Dentistry, Seoul National University Dental Hospital, Seoul National University, Seoul, Korea; 4 Graduate student, Department of Prosthodontics & Dental Research Institute, School of Dentistry, Seoul National University Dental Hospital, Seoul National University, Seoul, Korea; 5 Department of Prosthodontics, Seoul National University School of Dentistry, 101 Daehak-ro, Jongno-gu, Seoul, South Korea

**Keywords:** Contact adjustment, Dental prosthesis contact, Diamond strip, Proximal contact

## Abstract

**Background:**

Adjustment of proximal contact tightness in posterior crowns is critical for preventing food impaction and ensuring patient comfort. This study aimed to determine whether a conventional stone-based approach or a newly introduced diamond strip resulted in more efficient and predictable proximal contact adjustment in zirconia (Zr) and porcelain-fused-to-metal (PFM) crowns, as well as to assess whether clinician experience affects the outcome.

**Methods:**

To calculate the mean contact strength (baseline data), the tightness of the proximal connections of clinical crowns from 74 anonymous individuals was measured beforehand. Four working casts (containing premolars, first molars, and second molars) were made using commercially available resin die material. First and second molars were prepped, and metal crowns were cemented on the first molar to prevent errors in proximal contact correction between the first and second molars. Zr (*n* = 32) and PFM (*n* = 32) crowns on second molars were positioned 0.5 mm higher than the acceptable crown margin due to excessively tight proximal contacts. The tightness of these contacts was subsequently adjusted until the crowns were seated on the correct margin. Contact force (N) was measured using a force gauge placed between the adjusted crown and its adjacent tooth, and the time (chair time) necessary for proximal contact adaptation utilizing green stones with silicone polishers, and diamond strips was compared based on clinicians’ different experiences. The Mann–Whitney U test was used to assess differences in force and time between the groups.

**Results:**

The mean contact strength of clinical crowns was 4.55 ± 0.89 N. For the more experienced clinician, contact force of the green stone–adjusted restorations was higher than that of the diamond strip–adjusted restorations. The less experienced clinician required a longer duration to adjust with a green stone than with diamond strips, while the more experienced clinician showed similar adjustment times with both tools.

**Conclusions:**

The conventional way of achieving reasonable contact tightness using stones and silicone polishers may be influenced by clinicians’ experience. To shorten adjustment time, untrained practitioners can employ diamond strips.

## Background

The tooth is stabilized by the contact between the opposite and adjacent teeth [1]. The masticatory forces exerted on the teeth by the opposite teeth are transmitted to the adjacent teeth and periodontium through the interproximal contact areas. This redistribution of force provides an efficient mechanism for protecting the teeth and periodontium against trauma [2]. Furthermore, excessive occlusal loading caused by parafunctional masticatory movements, such as clenching and bruxism, can be relieved through proper proximal contact between the teeth and supporting tissues. Hence, ideal proximal contact areas and tightness play a vital role in maintaining the integrity of the dental arch.

The tightness of proximal contacts is defined as the resistance to separation of the contact areas during function [2]. Excessive pressure on the proximal contact between the teeth can cause wedging of teeth and undesirable tooth movement, resulting in crowding and repositioning of teeth that can change the occlusion and jaw position [3–8]. A weak or slightly open contact can result in food impaction and pain. Food impaction can lead to dental caries [4], halitosis, periodontal disease, or drifting of teeth [5] Thus, the restoration of proper proximal contact is crucial for occlusal rehabilitation [11–14]. 

The size, point, and degree of firmness of the proximal contact area depend on the anatomical contours of the adjacent proximal surfaces and the point of contact between the mesial and distal sides of the tooth. Achieving adequate tightness of the proximal contact in tooth restorations, which is determined by the snap entry of dental floss that passes through the contact surface with adjacent teeth, might be clinician dependent and difficult [9]. Others report that a metal shim stock is considered an accurate method to determine the tightness of the proximal contacts [1, 10–12] Therefore, clinicians typically use the floss snap method or shim stocks to determine the tightness of the proximal contacts.

The tightness of the proximal contact is considered excessive if the dental floss cannot penetrate the contact surface or tears during insertion; in contrast, the tightness is considered insufficient if the floss passes the contact surface without resistance. Although passing the floss through the proximal contact with a sense of resistance is the most conventional method for evaluating appropriate contact tightness clinically which is widely used, it cannot detect detailed changes and discrepancies in the tightness of the proximal contact that may vary among clinicians [13]. 

Therefore, a convenient method to achieve an appropriate contact strength repetitively must be established to simplify dental restorations. Clinicians have used stones or silicon to adjust the tightness of the proximal contacts; however, this can be dependent on the proficiency of the clinicians. Furthermore, most clinicians have had experiences with open contact while adjusting tight contact surfaces. The diamond strip that offers a quick and similar amount of adjustment each time, regardless of clinician-related factors, was introduced recently to resolve this inconsistency [14]. 

In this study, two methods of adjusting the tightness of proximal contacts—conventional method using stones and the recently introduced diamond strips—were compared in terms of the time and force of proximal contact, according to the proficiency of clinicians. The appropriate tightness of proximal contacts as references was determined in advance from anonymous clinical crowns before to delivery. The restoration of a crown prosthesis was simulated using two different materials: zirconia (Zr) and porcelain-fused-to-metal (PFM). The null hypothesis of this study was that there would be no difference in the time required to adjust the proximal contacts and contact force based on clinical proficiency with the use of the two adjustment methods (diamond strips or green stones with polishers).

## Methods

**Fig. 1:**
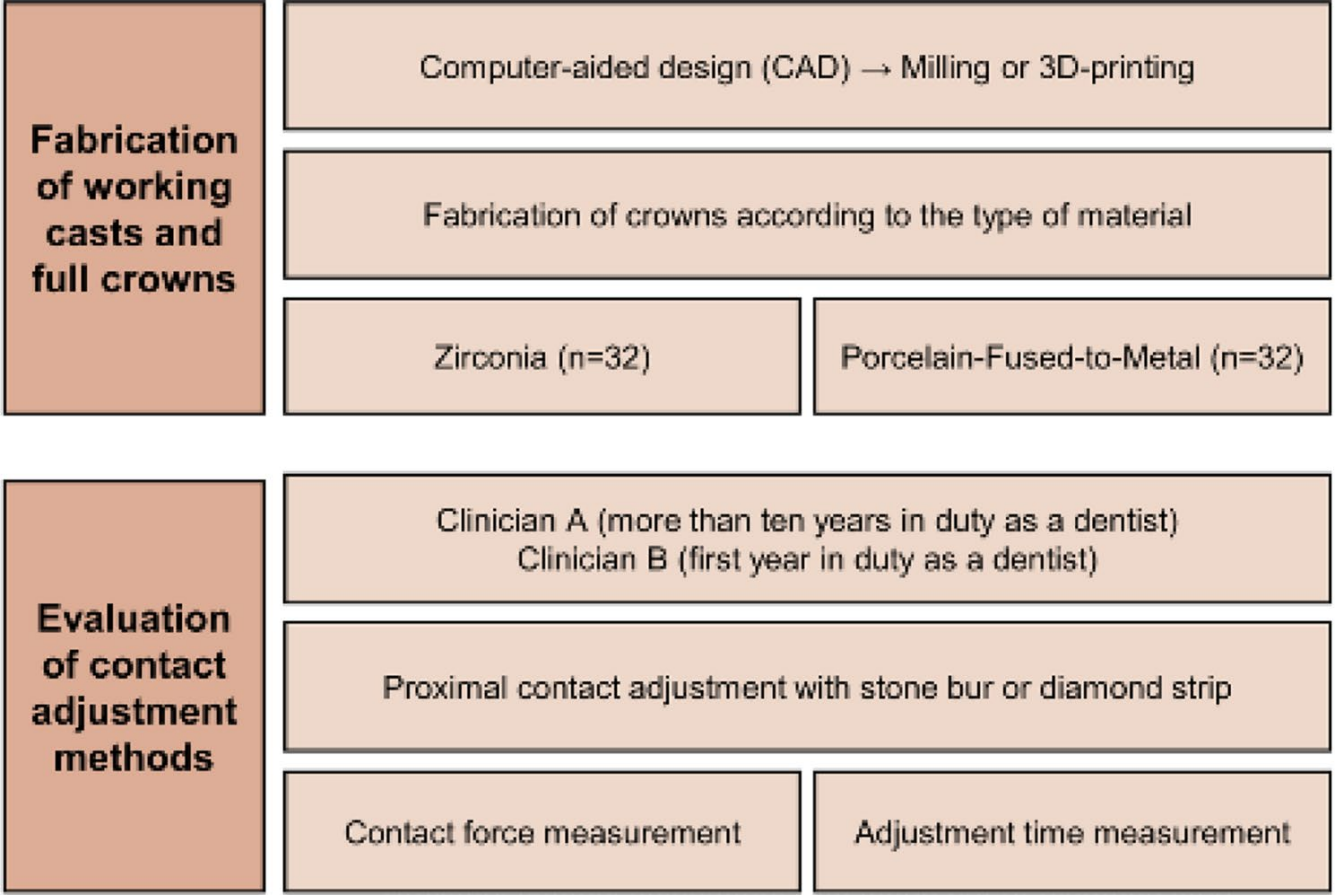
Simplified illustration of the study’s experimental process

**Fig. 2:**
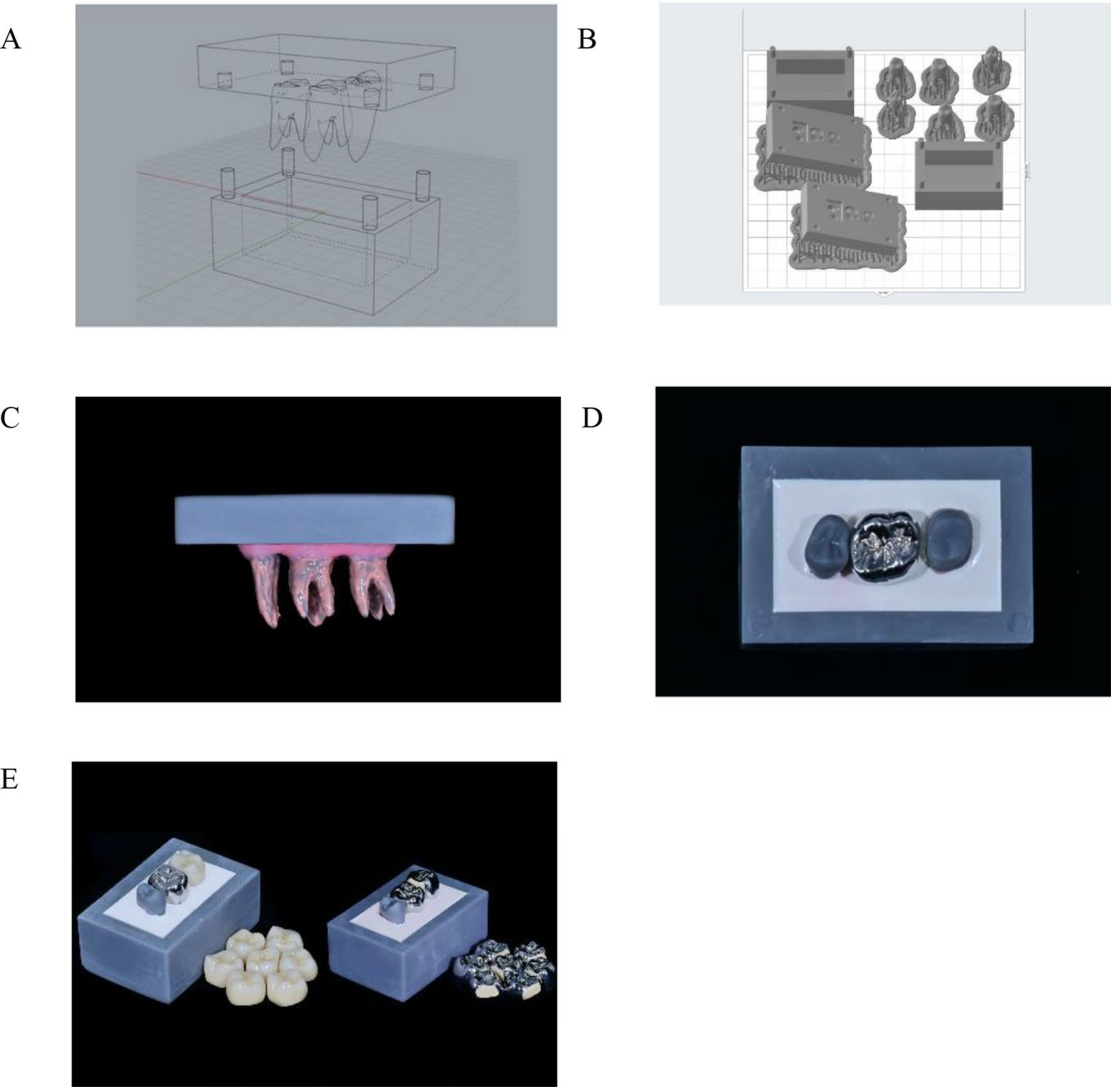
Fabrication of working casts and full crowns. **A** Computer-aided design of the working cast. **B** fabrication of the positioning jigs for the correct placement of the specimens. **C** root and simulated periodontal ligament of the specimens. **D** 3D-printed working cast. **E** fabricated full crowns with two different materials (zirconia and porcelain-fused-to-metal)

The experimental process used in this study is illustrated in Fig. 1. Computer-aided design (CAD) and three-dimensional (3D) printing technology were used to design and fabricate the working casts and experimental crowns. The right maxillary upper second premolar and first and second molars of a commercially available cast (B2-306; Nissin Dental) were used as abutments and scanned using a desktop scanner (T500; MEDIT Corp.). Four second premolars, four first molars, and four second molars of working casts were manufactured using a 3D printer (Form2; Fromlabs) with polyurethane resin die material (Polyurock; Metalor Technologies). The first and second molars of working casts were prepared for crowns. Adjustment of mesial contact area in crowns of second molars might occur the risk of damaging the distal surfaces of first molars manufactured with resin. To prevent those problems, first molars were prepared and cemented with Chrome-Cobalt metal crowns using resin cement (rely X universal resin cement, 3 M Corp.), shown in Fig. 2.

The originally scanned data of the first and second molars were also superimposed and replaced with those of the prepped teeth using CAD (Geomagic Control X; 3D Systems) to design a positioning jig. A positioning jig and a rectangular box to stabilize the uniform position of the teeth (4 premolars and 8 molars of the 4 groups) were also fabricated with a 3D printer. Twelve 3D-printed teeth of working casts were fixed in advance using the fabricated positioning jig (Fig. 2). Silicone impression material (Examix Fine; GC) was applied to the root of the tooth at a thickness of 0.25–0.3 mm to simulate the periodontal ligament. Four working castings with two molars and one premolar each were manufactured (Fig. 2). Clinicians used two working casts each, which were replaced following green stone and diamond strip adjustment of 16 crowns of Zr or PFM materials to reduce errors.

Thirty-two Zr crowns for second molars were designed using CAD software (Exocad; Darmstadt) and fabricated with milling machine (Craft 5X; DOF) with Zr block (Smartblock; Yesbio). These Zr crowns had overly large proximal surfaces that were seated 0.5 mm higher than the correct position of the margin for the room of adjustment of contact surface. Thirty-two PFM crowns were also designed using CAD software and fabricated with a 3D printing machine (Concept Laser Mlab; Concept Laser GmbH). Their mesial surfaces were removed by 1.2 mm of the Co-Cr material to enable the further addition of porcelain. The printed Co-Cr (Starbond CoS powder; Scheftner Dental Alloys) crowns were cleaned in distilled water using an ultrasonic cleaner (BioSonic UC50; COLTENE) for 10 min and subsequently rinsed with ethyl alcohol for 10 min to remove surface residue. After oxidation, the samples were blasted with 50 μm Al_2_O_3_ particles (Eazimill A11; Vericom) for 5 s under 4 atm pressure from a distance of 5 cm. Opaque and body porcelain (Willi-Geller creation powder; Impulsedent Australia) were applied according to the manufacturer’s directions to fabricate an overly large PFM proximal surface until the crowns were seated 0.5 mm higher than the correct position of the margin. The samples were subsequently fired in a ceramic furnace (Programat p500; Ivoclar Vivadent) according to the manufacturer’s specifications.

**Fig. 3:**
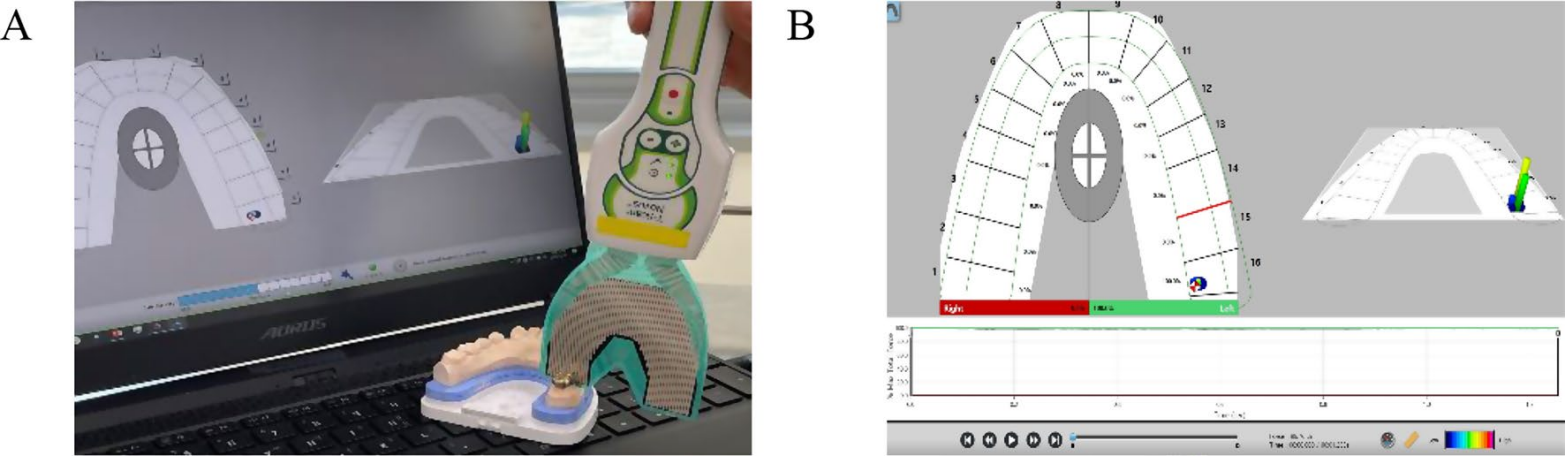
Evaluation of proximal tightness with T-scan sensor **A**, measurement of the clinical crowns as references. **B**, screen image of the examination of contact strength with T-Scan

**Fig. 4:**
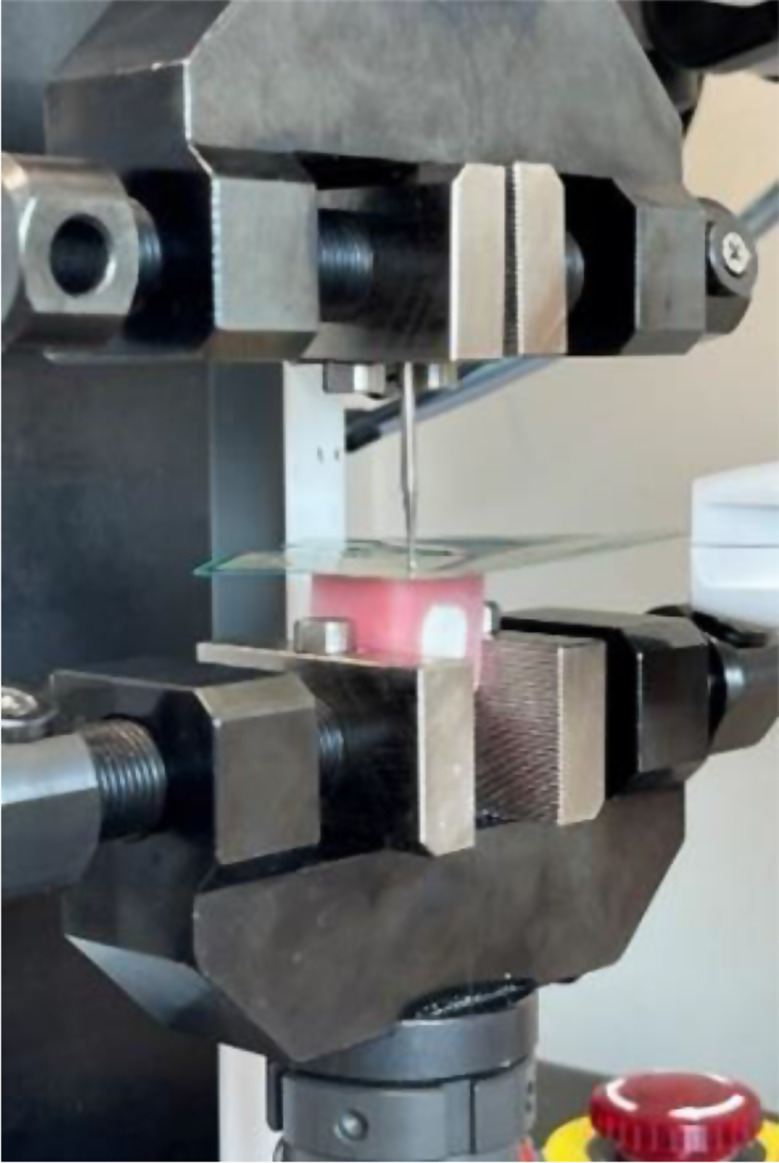
Universal Test Machine with T-scan sensor

The proximal contacts of the clinical crowns seated in the casts obtained from patients were assessed after chairside adjustment using the dental floss snap technique to quantify the inter-proximal contact force as a reference for proper tightness of the proximal contact (*n* = 74). The average contact force which was a relative unit (raw force) was derived using Digital Occlusal Analysis equipment (T-scan; Tekscan) as shown in Fig. 3. The calculated contact force was extracted using the American Standard Code for Information Interchange (ASCII) module. A universal testing machine (UNITEST M1; TEST ONE) with a spherical bur (diameter = 2.2 mm) approximately the size of the proximal contact point was used to convert this relative force to a quantified Newton (N) force (Fig. 4). The N force chart created by universal testing machine was compared with the relative force retrieved from the T-scan sensor, and the ratio of the two values was calculated. Therefore, the calculation formula was derived.

**Fig. 5:**
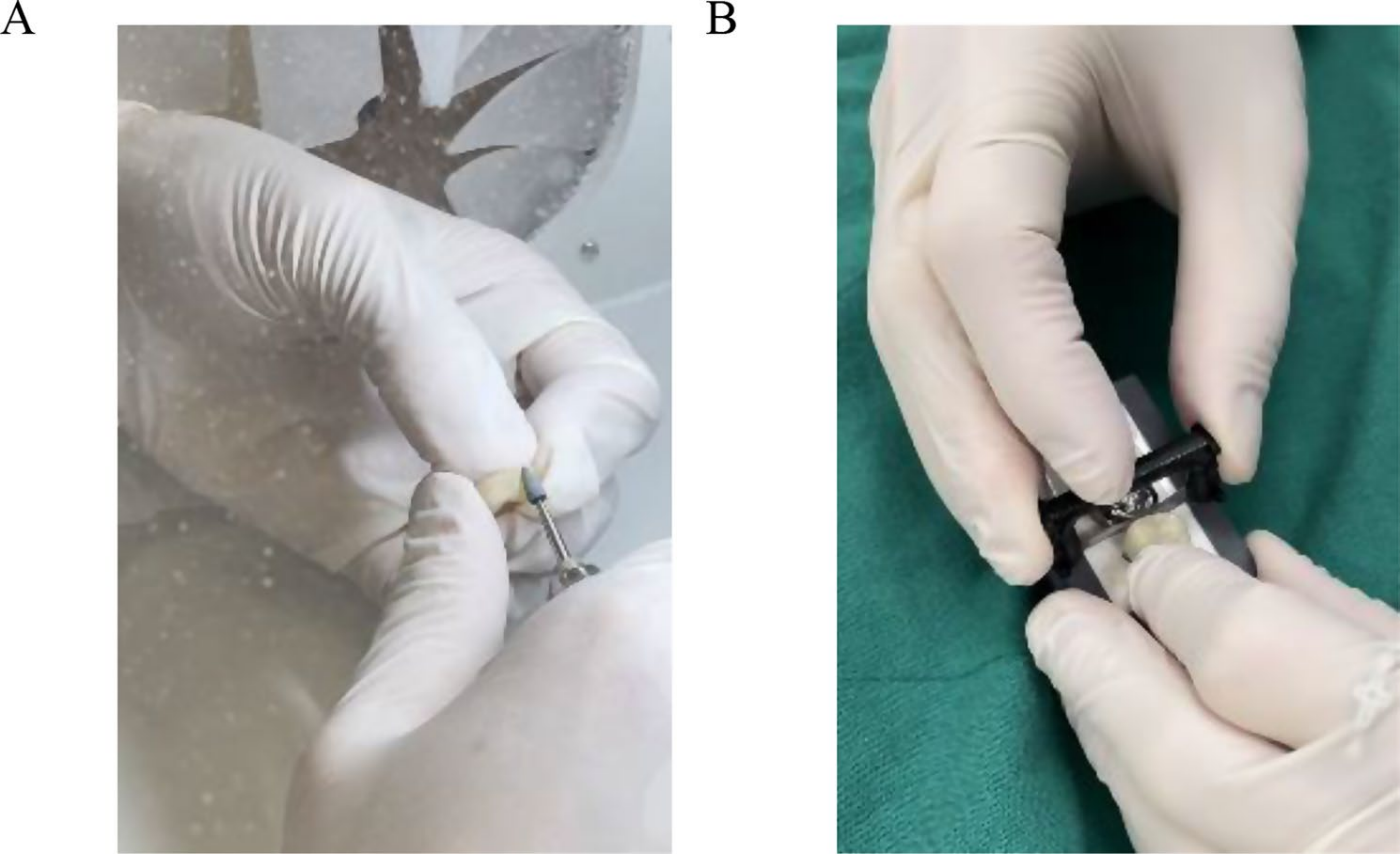
Proximal contact adjustment. **A** Adjustment of the restoration using green stone bur. **B** Adjustment of the restoration using diamond strip

The 64 crown (32 Zr crowns and 32 PFM crowns for second molars) specimens were divided into 8 groups: SZA (Stone bur, Zr, Clinician A, *n* = 8), DZA (Diamond strip (IPR strip; contacEZ), Zr, Clinician A, *n* = 8), SZB (Stone bur, Zr, Clinician B, *n* = 8), DZB (Diamond strip, Zr, Clinician B, *n* = 8), SPA (Stone bur, PFM, Clinician A, *n* = 8), DPA (Diamond strip, PFM, Clinician A, *n* = 8), SPB (Stone bur, PFM, Clinician B, *n* = 8), DPB (Diamond strip, PFM, Clinician B, *n* = 8). Two Clinicians adjusted the contact surfaces of eight Zr crowns each with both Green stone (Dura-green stones; Shofu) and polishers (Zr polishers for Zr; Ceramiste polishers for PFM, Shofu) and eight Zr crowns each with the diamond strips (black diamond strips and gray polisher strips). The same procedures were proceeded for 32 PFM crowns (Fig. 5).

The specimens were positioned 0.5 mm higher than the correct position of the crown margin owing to the excessively tight proximal contacts. The tightness of the contacts was subsequently adjusted until the crowns were seated on the correct margin, then clarified using the dental floss snap technique with intermediate resistance to floss (Oral-B essential mint floss; Proctor and Gamble) passage, as specified in the study’s experimental criteria. Standardized training and clear protocols were provided to minimize inter-clinician variability. And before the experiment, two clinicians agreed on the degree of resistance force to the passage of floss in models, and the shim stock method was not employed because it was difficult for an inexperienced practitioner. The duration and force required to adjust the tightness of the proximal contacts until all specimens were perfectly seated for Clinicians A and B, who were classified according to their proficiency (Clinician A: more than ten years of experience as a dentist, Clinician B: first year of experience as a dentist), were investigated. Differences in the time and contact strength were determined between the two clinicians. The entire process of contact adjustment was recorded to measure the duration. The tightness of the proximal contacts was also measured using T-scan software like previous reference data. The T-Scan Novus Dental Sensor was placed between the crowns of the upper right first (Metal crowns) and second molars (Zr or PFM crowns), and the tightness of the proximal contacts was measured when the second molar crown was seated at the correct marginal location. The T-scan sensitivity was set to an intermediate level at the time of recording, similar to the reference value which is gained from 74 clinical crowns. The values measured using the ASCII module were extracted and the average values were calculated. The ratio obtained from the previously described procedure was used to convert the relative raw contact forces into N forces (both contact force data from the 74 anonymous clinical crowns and 64 experimental groups).

Statistical analyses were performed using SPSS (IBM Statistics, version 25.0; IBM Corp, Armonk). Statistical significance was set at *p* <.05. The Shapiro–Wilk test was used to assess the normality of the data. Since the data did not follow a normal distribution, non-parametric tests were employed. The Mann–Whitney U test was used to assess the force and time differences between the groups.

## Results

In this study, the contact strength of the 74 anonymous clinical crowns was assessed prior to delivery after adjusting for proximal tightness with adjacent teeth. The following conversion formula was derived and used in the calculation:

$$\:Fa\hspace{0.17em}=\hspace{0.17em}C\:\times\:\:Fr$$

where Fa is the actual contact force, C is a constant, and Fr is the raw contact force.

The conversion constant C was determined as 1raw = 0.0523 N through calibration experiments. This constant was consistently used to convert raw contact force (*Fr*) into actual contact force (*Fa*) for all 74 clinical crowns and 64 experimental groups. The contact strength of reference data varied from 2.42 to 6.38 N, and the mean value was reported as 4.55 ± 0.89 N.

Table 1 presents the contact forces of all specimens for the two clinicians in a contact experiment with 64 (8 of 8groups each) second molar crowns. There was a significant difference only between Clinicians A and B adjusting Zr crowns with green stone (*P* =.015).

**Table 1:** Contact force differences between the two clinicians

	Materials	Adjustment tool	Clinician A	Clinician B	df	*P* value
Force (N)	Zr	Diamond strip	4.43 [4.26–5.11]	4.02 [3.37–5.03]	7	0.328
		Green stone	5.59 [4.93–5.96]A	4.59 [3.57–5.20]B		0.015 *
	PFM	Diamond strip	4.08 [3.74–4.59]	4.21 [3.57–4.36]		0.878
		Green stone	4.77 [4.19–5.29]	4.53 [4.32–4.86]		0.442

df, degrees of freedom

Interquartile ranges [1st quartile, 3rd quartile] are in parentheses

Different uppercase letters indicate statistically significant differences by Mann-Whitney U test (*p* <.05) (A > B)

Zr, zirconia; PFM, porcelain-fused-to-metal

**Fig. 6:**
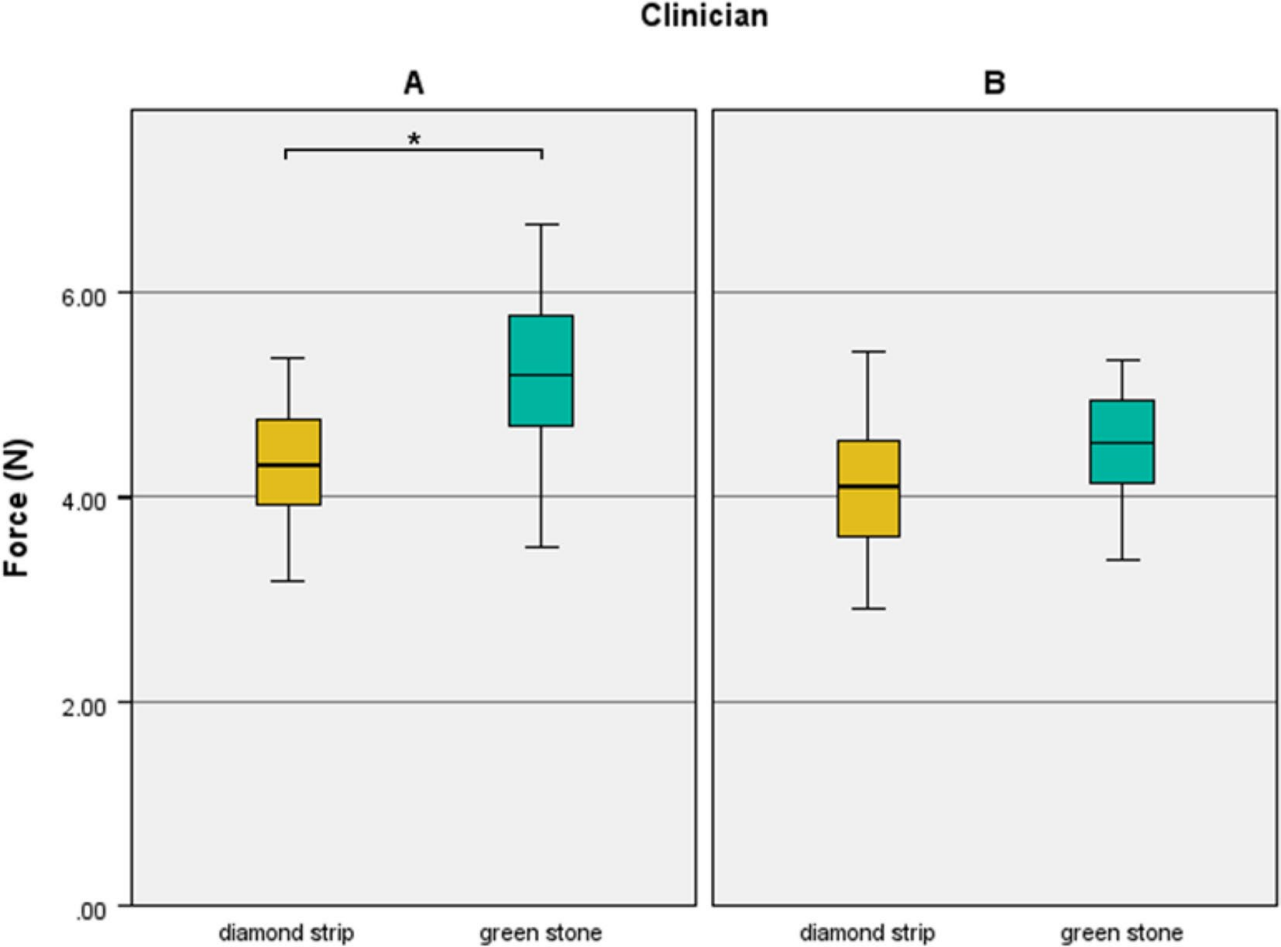
The contact force differences according to adjustment method

Figure 6 shows that the contact force of the green stone-adjusted restorations was higher than that of the diamond strip-adjusted restorations in the clinician A, who had more expertise. Additionally, proximal contact adjusted with diamond strips (4.25[3.76–4.67] N), regardless of clinician or material, were substantially less tight than those adjusted with green stones (4.83[4.30–5.38] N) (*P* =.005).

**Table 2:** Contact adjustment time differences between the two clinicians

	Materials	Adjustment tool	Clinician A	Clinician B	df	*P* value
Time (s)	Zr	Diamond strip	75.50 [59.00-85.25]	76.50 [37.25–118.50]	7	0.878
		Green stone	64.50 [62.00–78.00]B	251.50 [195.25-266.25]A		< 0.001 *
	PFM	Diamond strip	86.00 [67.75–110.00]	88.00 [76.25–99.75]		0.798
		Green stone	72.50 [49.75–84.50]B	94.00 [88.00-131.00]A		0.003 *

df, degrees of freedom

Interquartile ranges [1st quartile, 3rd quartile] are in parentheses

Different uppercase letters indicate statistically significant differences by Mann-Whitney U test (*p* <.05) (A > B)

Zr, zirconia; PFM, porcelain-fused-to-metal

**Fig. 7:**
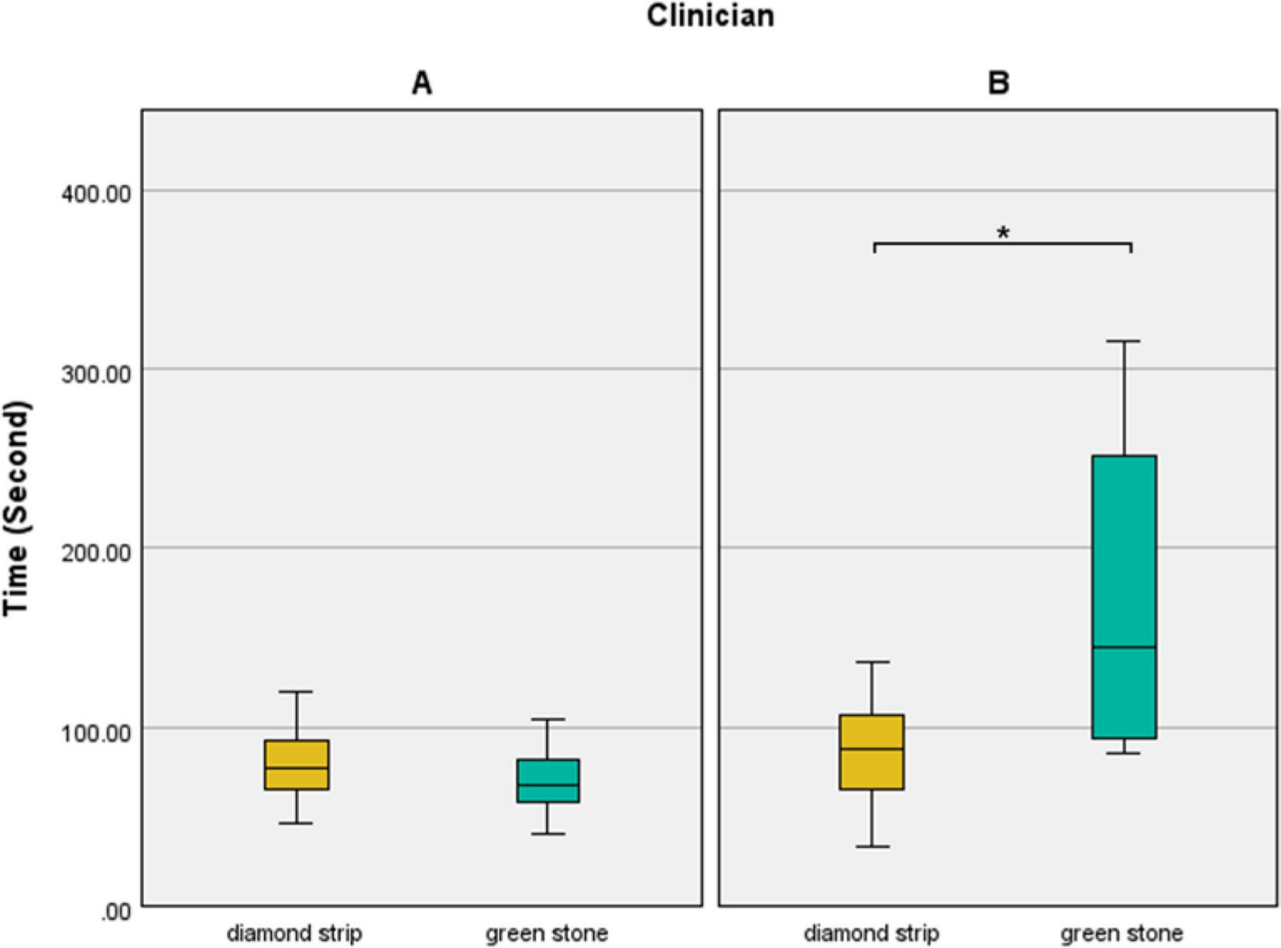
The time differences according to adjustment method

The time required for adjusting the proximal contacts differed significantly between the clinicians in second molar crowns. Table 2 presents the duration required for contact adjustment in each group. Clinician A demonstrated a shorter adjustment time than Clinician B in each group; however, statistically significant differences were observed between the clinicians only in the groups using green stone (Zr, *P* <.001; PFM, *P* =.003). As shown in Fig. 7, Clinician B required a longer duration to adjust with a green stone and polisher than with diamond strips, while Clinician A showed similar adjustment times with both adjustment tools. Furthermore, diamond strips, regardless of clinician or material, required less adjustment time (81.50[65.25–99.75] s) than green stone (88.00[67.00-162.25] s), but the difference was not statistically significant (*P* =.140).

The intraclass correlation coefficient (ICC) was used to analyze the reliability between two clinicians. The analysis yielded a Cronbach’s alpha value of 0.718, confirming satisfactory reliability.

## Discussion

The proximal contact force adjusted by the two clinicians indicated that Clinician A (who had more clinical experience) achieved greater tightness of the contact in each group; even the Zr group with green stone showed considerably greater contact force than that of those of clinician B (Table 1). A considerable variation was observed in the time consumption between the two practitioners with varying clinical expertise. However, substantial time differences were observed only in the groups that used green stone to adjust the tightness of the proximal contacts (Table 2). Thus, the null hypothesis of this study was rejected. These findings demonstrate that clinical proficiency affects adjustment time when greenstone is used, which represents the standard method of proximal contact, but not when diamond strips are used. Thus, practitioners who are not experienced in adjusting the proximal contact can reduce chairside adjustment time by using diamond strips.

Previous studies reported large intra- and inter-individual variation in the tightness of the proximal contacts, and the authors determined that it is difficult to define “optimal” proximal contact tightness [2–3, 12, 15–16]. The patients did not report any discomfort when a tight contact was reconstructed, and some patients only reported inconvenience when a loose or open contact caused food impaction, including periodontal problems [13–15]. Patients and clinicians may not detect any issues with existing contact tightness until periodontal or other problems become apparent; thus, effectively modifying the tightness of the proximal contacts is a difficult and time-consuming process for clinicians, particularly those with limited experience.

Diamond strips adjust the tightness of the proximal contact by sawing a constant amount of prosthetic material between the new restoration and adjacent teeth in the mouth; consequently, this method is less affected by clinical proficiency as practitioners can directly visualize whether the new restorations are seated. The results of the present study demonstrated that the diamond strip adjustment approach saves more time than the traditional method, which uses green stones (Table 2). As a result, employing diamond strips may assist practitioners in achieving correct proximal contact more easily and quickly than traditional adjusting instruments, such as stone burs.

In this study, the proximal contact strength of 74 adequately adjusted (using conventional method; floss snap technique and shim stock check) clinical crowns was tested beforehand and utilized as the reference. The substantial standard deviations of the tightness of the proximal contacts from the reference data and the experimental group support the premise that determining the optimal tightness of the proximal contacts is difficult. Despite this limitation, a range of tightness of the proximal contact from anonymous clinical crowns was calculated to compare the measured number with the tightness of the proximal contacts of the experimental group. As a result of using a diamond strip, both clinicians in this trial demonstrated tightness of the proximal contacts within the range of the measured reference data, as the clinician can visualize where the restoration is being adjusted. However, only Zr group with the trained practitioner utilizing the usual adjustment method with green stone had a greater mean contact force than the reference data. Overall, it indicates the clinical challenges and biases that practitioners have when adjusting the tightness of the proximal contact and evaluating it with a floss snap. Furthermore, the tightness of the proximal contacts adjusted using green stones, regardless of the clinician or material, was considerably higher than those adjusted with diamond strips (*P* =.005). When diamond strips are used, a constant amount of proximal contact is removed depending on the number of sawings; nevertheless, for green stones, clinicians adjust restorations based on instinct, sometimes less, sometimes more although the agreement and practice of resistance in this experiment. It demonstrates that the conventional proximal contact adjustment method using stones might be clinician reliant, emphasizing the complexity of this procedure.

Almalki et al. reported that 66% of dental clinicians aim to create a proximal contact naturally, yet 18% achieve an open proximal contact, and 16% achieve a tight proximal contact [15]. This finding is consistent with the results of the study by Oh et al., who discovered that 58.4% of practitioners create a proximal contact normally, whereas 12.6% create tight proximal contact [16]. Thus, dental practitioners have a bias when it comes to the tightness of the proximal contacts. In this study, Clinician A was also more likely to create tight proximal contacts than the reference data as she had received several complaints from patients over the years. In South Korea, most people use high-fiber foods on a daily basis, resulting in frequent food lodgment in the proximal contact which causes pain; under these circumstances, the clinician may incline to build tighter proximal contacts than appropriate proximal contacts as they acquire experience. This finding is in line with the findings of the study by Boice et al. which compared the tightness of the proximal contacts between natural teeth and prostheses and found that prostheses had tighter proximal contacts than natural teeth [12]. In this study, the contact force between adjustment tools regardless of clinician and material, was significantly less in diamond strip groups, (*P* =.005) indicating that diamond strip method might prevent biased clinicians from causing the adjacent teeth drift and the modify occlusion by making contact excessively tight.

Excessively tight proximal contacts cause pain and discomfort to patients, thereby necessitating adjustment. The contact tightness should not be so tight that it causes tooth wedging and movement. However, clinicians may make mistakes in order to finish restorations with appropriate contact tightness. Previously, there was no method to modify the tightness after the final cementation of the prosthesis. However, diamond strips can be used when prostheses are seated in the mouth; thus, dentists can now adjust the proximal contact even after cementation, which can be advantageous. Also, it is widely known that after the use of a green stone where is usually a need to polish the area, that might occur open contact due to the amount of porcelain removed. This may occur less frequently with diamond posher strips.

The proximal contact strength is not a constant value and varies with posture and occlusion [1]. As a result, even when clinicians carefully adjust the tightness of the proximal contacts, patients may report tightness of the proximal contacts following treatment. In such circumstances, a diamond strip can be used to modify the contact. However, clinician should be careful while using diamond strips, since their thickness (approximately 65–75 μm) exceeds that of commercially available shim stock (approximately 13–30 μm). As a result, it is difficult for clinicians to insert diamond strips between restorations and adjacent teeth depending on the periodontal health and the mobility of adjacent teeth. Therefore, clinicians might frequently encounter the need of a wedge for the use of a diamond strips. Even when diamond strips are inserted between prostheses and adjacent teeth, there is a possibility that strips migrate to adjacent teeth for a short period of time, causing discomfort to patients.

There are different opinions regarding the measured values of the interproximal space. The interproximal contact space at rest measured using a charge coupled device microscope was found to be 3–21 μm [17]. Campagni et al. reported that the proximal contact tightness is appropriate when the shim stock of approximately 13 μm passes though the proximal contact with slight resistance and two shim stocks cannot pass, indicating that the proximal contact is under 30 μm [9]. Boice et al. also indicated that most adults have an interproximal contact space of approximately 13 μm through which a shim stock can pass [12]. Guichet et al. suggested that optimal interproximal contact space of implant restoration is approximately 8 μm, with tight space extending up to 90 μm [18] Miura et al. reported that a 50-µm metal strip can pass through interproximal contact space with resistance; however, a 110-µm strip cannot [19]. These varying widths of the interdental gap reflect the difficulties in adjusting the tightness of the proximal contacts as each patient is accustomed to different widths and forces of proximal contact. Furthermore, it is speculated that the proper interproximal gap between adjacent teeth may be affected by the dietary habits of individuals from different cultures. This notion should be examined further in future studies.

A previous study demonstrated that the contact strength varies from 0.72 to 2.44 N [20], whereas another study reported that the contact strength between natural teeth at rest is 0.22–2.20 N and that on clenching is 1.48–23.04 N force [17]. The measured contact strength shows such diversity that it cannot be defined and is rarely known. In this study, the measured contact strength of the 74 anonymous clinical crowns, after correction for proximal tightness with adjacent teeth before delivery, varied from 2.42 to 6.38 N, and the mean value was reported as 4.55 ± 0.89 N. This finding is consistent with that of the study by Dörfer et al., [2] which indicated that proximal contact strength is considerably influenced by location, tooth type, chewing, and time of day. The contact strength and tightness of the new restoration should be adequate to maintain healthy and comfortable adjacent and abutment teeth, although it still tends to remain in the clinical region, depending on the clinician’s expertise and experience. However, diamond strips can assist inexperienced practitioners with direct observation to achieve proper contact tightness and force, thereby saving time, effort, and confusion.

Another finding of this study was that inexperienced clinicians took considerably longer time to adapt harder restoration materials, indicating that Zr required more time. According to previous studies, the hardness of Zr and porcelain in PFM differ vastly. (21–22) As a result, the time required can vary depending on the instrument the clinician uses as an adjustment tool for adjusting the proximal contact. However, as the properties of various materials have already been determined, competent professionals can vary the adjustment volume and force while using conventional green stones, resulting in quicker adjustment time. Clinician B required a longer duration to adjust prostehses by a green stone with a silicone polisher than diamond strips, while clinician A showed similar adjustment time in both materials (Table 2). Less experienced clinicians appeared to be doubtful about the amount of removing by stone bur because they were afraid to make it open contact, resultantly, using stone burs can make adjustment time longer.

A disadvantage of this study is that floss snapping was used to assess contact tightness, which may be subjective. There are limitations that it is not assumed that PFMs experienced pyro-plastic flow which could have affected the actual amount remaining and altered force transmitted when force was measured [23]. And the concept of a simulated periodontal ligament is controversial and there is no consensus on how to do it. In this study if the T-Scan test, or the floss test, was performed without it, the results would be different. and they would not be subjected to the variations of differences in the thicknesses of the silicone (despite the effort required for consistent silicone thickness) or concerns of how the silicone relates to a PDL. Furthermore, sample size could not be calculated using statistical methods, indicating that this study is a pilot study. Nonetheless, this study sheds light on the critical significance of clinical proficiency in adjustment time and the impact of various adjustment approaches and more research is needed.

## Conclusions

The average contact force in South Korea is assumed to be approximately 4.5 N. The conventional way of achieving reasonable contact tightness using a low-speed motor and stones may be affected by clinicians’ proficiency and experience. Therefore, the use of diamond strips, which reduces a constant amount of contact with each sawing motion, while enabling direct observation of the prostheses, can be used as an alternative by inexperienced practitioners.

## Data Availability

The datasets used and/or analyzed during the current study are available from the corresponding author on reasonable request.

## Abbreviations

Zr: Zirconia
PFM: Porcelain-fused-to-metal
